# Supplementary material for: Phenolic Compounds Content Evaluation of Lettuce Grown under Short-Term Preharvest Daytime or Nighttime Supplemental LEDs
Source: Plants (Basel). 2022 Apr 21;11(9):1123. doi: 10.3390/plants11091123 (PMC9105848; doi:10.3390/plants11091123)
Supplement: Supplementary file 1 [file plants-11-01123-s001.zip › plants-1659833-supplementary.pdf]

Table S1. Effect of short-term daytime supplemental LEDs to HPS lighting on phenolic compounds content in lettuce cultivated in a greenhouse.

Phenolic Compounds	HPS	400 nm	455 nm	455 + 530 nm	530 nm	660 nm
Caffeic a.	0.042 b	0.036 b	0.048 ab	0.048 ab	0.016 b	0.097 a
Chicoric a.	3.228 a	3.747 a	3.036 a	4.044 a	3.066 a	2.347 a
Chlorogenic a.	0.927 a	1.026 a	1.071 a	1.101 a	0.747 a	0.983 a
Gallic a.	0.039 a	0.027 a	0.028 a	0.026 a	0.023 a	0.023 a
o-coumaric a.	0.090 a	0.132 a	0.049 a	0.047 a	0.044 a	0.133 a
p-coumaric a	0.045 a	0.034 a	0.027 a	0.020 a	0.032 a	0.019 a
Protocatechuic a.	0.124 a	0.081 a	0.077 a	0.048 a	0.090 a	0.118 a
Rosmarinic a.	0.345 a	0.336 a	0.626 a	0.494 a	0.516 a	0.317 a
Apigenin	0.588 a	0.360 a	0.541 a	0.251 a	0.548 a	0.305 a
Epicatechin	0.222 a	0.256 a	0.103 a	0.139 a	0.117 a	0.319 a
Kaempferol	0.027 a	0.025 a	0.020 a	0.023 a	0.029 a	0.021 a
Myricetin	0.085 a	0.062 a	0.070 a	0.046 a	0.068 a	0.046 a
Quercetin	0.033 a	0.108 a	0.020 a	0.107 a	0.043 a	0.084 a
Rutin	0.034 b	0.037 b	0.036 b	0.038 b	0.018 b	0.186 a
Total	5.827 a	6.267 a	5.753 a	6.432 a	5.355 a	4.997 a

Individual phenolic compound content is presented as mg g^{-1} in dry plant matter. Means with different letters are significantly different at the $p < 0.05$ level by Tukey's honestly significant difference test.

Table S2. Effect of different seasons on phenolic compounds content in lettuce cultivated in a greenhouse under short-term daytime supplemental LEDs to HPS.

Phenolic Compounds	Spring	Autumn
Caffeic a.	0.041 a	0.055 a
Chicoric a.	2.339 b	4.150 a
Chlorogenic a.	0.855 b	1.096 a
Gallic a.	0.034 a	0.021 b
o-coumaric a.	0.042 b	0.123 a
p-coumaric a	0.042 a	0.017 b
Protocatechuic a.	0.137 a	0.042 b
Rosmarinic a.	0.655 a	0.223 b
Apigenin	0.650 a	0.215 b
Epicatechin	0.094 b	0.292 a
Kaempferol	0.028 a	0.021 b
Myricetin	0.086 a	0.039 b
Quercetin	0.040 b	0.091 a
Rutin	0.020 b	0.096 a
Total	5.064 b	6.480 a

Individual phenolic compound content is presented as mg g^{-1} in dry plant matter. Means with different letters are significantly different at the $p < 0.05$ level by Tukey's honestly significant difference test.

Table S3. Effect of short-term nighttime supplemental LEDs to HPS lighting on phenolic compounds content in lettuce cultivated in a greenhouse.

Phenolic Compounds	HPS	400 nm	455 nm	455 + 530 nm	530 nm	660 nm
Caffeic a.	0.129 a	0.130 a	0.144 a	0.078 a	0.139 a	0.094 a
Chicoric a.	2.376 a	1.401 a	1.865 a	2.895 a	1.032 a	1.011 a
Chlorogenic a.	1.847 ab	1.770 ab	1.188 b	3.257 a	1.187 b	1.403 b
Gallic a.	0.035 a	0.033 a	0.021 a	0.031 a	0.026 a	0.022 a
o-coumaric a.	0.247 a	0.213 a	0.053 a	0.067 a	0.075 a	0.051 a
p-coumaric a	0.013 a	0.024 a	0.015 a	0.028 a	0.014 a	0.021 a
Protocatechuic a.	0.111 a	0.072 a	0.057 a	0.084 a	0.060 a	0.092 a
Rosmarinic a.	0.052 a	0.369 a	0.580 a	1.162 a	0.229 a	0.388 a
Apigenin	0.200 a	0.239 a	0.150 a	0.304 a	0.153 a	0.129 a
Epicatechin	0.294 a	0.072 a	0.091 a	0.106 a	0.279 a	0.097 a
Kaempferol	0.007 a	0.008 a	0.009 a	0.010 a	0.007 a	0.016 a
Myricetin	0.029 a	0.039 a	0.023 a	0.025 a	0.028 a	0.018 a
Quercetin	0.015 a	0.011 a	0.011 a	0.012 a	0.017 a	0.014 a
Rutin	1.494 a	2.571 a	0.680 a	1.741 a	1.266 a	1.107 a
Total	6.849 ab	6.953 ab	4.886 b	9.799 a	4.513 b	4.463 b

Individual phenolic compound content is presented as mg g^{-1} in dry plant matter. Means with different letters are significantly different at the $P < 0.05$ level by Tukey's honestly significant difference test.

Table S4. Effect of different seasons on phenolic compounds content in lettuce cultivated in a greenhouse under short-term nightime supplemental LEDs to HPS.

Phenolic Compounds	Spring	Autumn
Caffeic a.	0.198 a	0.041 b
Chicoric a.	3.270 a	0.257 b
Chlorogenic a.	2.392 a	1.159 b
Gallic a.	0.039 a	0.017 b
o-coumaric a.	0.096 a	0.139 a
p-coumaric a	0.030 a	0.008 b
Protocatechuic a.	0.139 a	0.019 b
Rosmarinic a.	0.919 a	0.008 b
Apigenin	0.361 a	0.031 b
Epicatechin	0.247 a	0.066 b
Kaempferol	0.016 a	0.003 b
Myricetin	0.031 a	0.023 a
Quercetin	0.022 a	0.005 b
Rutin	0.076 b	2.877 a
Total	7.836 a	4.652 b

Individual phenolic compound content is presented as mg g^{-1} in dry plant matter. Means with different letters are significantly different at the $P < 0.05$ level by Tukey's honestly significant difference test.
